# Real-world face super-resolution based on generative adversarial and face alignment networks

**DOI:** 10.1038/s41598-026-37573-0

**Published:** 2026-02-20

**Authors:** Hebatallah Fathy, Mohamed Talaat Faheem, Reda Elbasiony

**Affiliations:** 1 Faculty of Engineering Tanta University, Computers and Automatic Control Engineering, Tanta University, Tanta, 37133 Egypt; 2 Egypt-Japan University of Science and Technology (E-JUST), Alexandria, 21934 Egypt

**Keywords:** Generative adversarial network (GAN), Face super-resolution, Facial recognition, Degradation kernel, Engineering, Mathematics and computing

## Abstract

Facial recognition performance is significantly limited when dealing with low-resolution face images, especially in real-world scenarios, due to the lack of precise knowledge about the degradation kernel. This research aims to enhance the resolution of real-world low-resolution face images by integrating a face alignment network into a semi-cycle generative adversarial network (GAN), which is conventionally known as face super-resolution. The proposed approach leverages the powerful capabilities of GANs to alleviate the domain discrepancy between real and synthetic images by introducing dual degradation pathways (forward and backward) that work collaboratively within a cycle-consistency learning framework. Additionally, a face alignment network is embedded within the GAN framework to refine the generated images by leveraging heatmap regression, which predicts the precise locations of facial landmarks. This allows our method to enforce structural consistency and preserve fine-grained facial details, such as the eyes, nose, and mouth, in the super-resolved images. As a result, the proposed method achieves significant improvements in generating high-resolution realistic face images. The experiments were conducted on both real-world and synthetic datasets; the results demonstrated the superiority of our method over existing approaches in generating high-resolution face images with exceptional degradation kernel and naturalness. Additionally, our method achieved the highest accuracy in face recognition and detection tasks, reflecting its capability to preserve essential identity features effectively, making it particularly well-suited for applications involving downstream facial analysis.

## Introduction

Face super-resolution (FSR) is a vital technique that reconstructs high-resolution (HR) face images from low-resolution (LR) counterparts, enhancing image quality and improving face recognition performance. This is particularly important in applications such as facial editing, security, and surveillance, where accurate face recognition is essential. However, the quality of facial details often deteriorates when images are captured at low resolution, diminishing the ability to extract meaningful facial details. The challenge becomes even more pronounced in real-world scenarios, where image degradation patterns are often unpredictable and inconsistent^1–3^, and^4^.

Face super-resolution (FSR) has advanced significantly, progressing from traditional techniques to deep convolutional neural networks (CNNs) and further to generative adversarial networks (GANs). Traditional FSR approaches are generally categorized into three types: local patch-based approaches^5,6^, and^7^, global image-based approaches^8,9^, and^10^, and hybrid approaches that integrate the benefits of both by maintaining global image coherence while preserving local details^11–13^, and^14^. However, these traditional handcrafted approaches frequently fail to adequately handle the diverse and complex degradations commonly encountered in real-world settings^15^.

Deep convolutional neural networks (CNNs) have recently exhibited substantial potential in advancing face super-resolution (FSR) techniques^16–19^ and^20^. These models primarily focus on learning a direct mapping function to restore high-resolution (HR) face images from their low-resolution (LR) forms. For evaluation purposes, LR images are often artificially created by applying artificial degradation techniques to HR images. However, in real-world scenarios, obtaining the corresponding HR images for the actual LR inputs is a significant challenge. This discrepancy leads to a considerable performance gap between synthetic training data and practical applications in real-world face SR tasks. To bridge this gap, some methods^21,22^, and^23^ have been designed to align the LR face images with the unpaired HR images of the same identity. Nevertheless, the process of face alignment is hindered by the limited availability of HR images in real-world contexts, which results in under trained face super-resolution models and further challenges the preservation of identity details.

In comparison to discriminative methods, generative CNNs, such as GANs^24^, have been utilized in several studies^15,21,25^^26^^27^,,, and^28^ to address blind face super-resolution (SR) under complex degradation conditions. To handle unknown real-world degradations, some generative CNNs^29^^30^^31^,,, and^32^ have adopted unsupervised face SR approaches, leveraging the concept of cycle-consistency developed for unpaired image translation tasks^33^. A notable example is LRGAN^29^, which incorporates a cycle learning framework for real-world face SR. It introduces two branches: a “learning-to-degrade” branch for simulating image degradation and a “learning-to-SR” branch for performing super-resolution. However, due to the significant disparity in identity information between unpaired LR and HR face images, the two branches in LRGAN^29^ achieve consistency primarily for HR images. As a result, the model struggled to effectively retain facial details and identity information for LR images.

The uncertain relationship between unpaired low-resolution (LR) and high-resolution (HR) face images makes it difficult for directional frameworks^29^ or fully-cycled bidirectional approaches^33^ to preserve identity information in real-world scenarios. To address this, SCGAN^34^ introduced the Semi-Cycled Generative Adversarial Network, which extends the bidirectional cycle-consistency framework^33^ with a more flexible design. SCGAN^34^ incorporates three branches: (1) a “learning-to-degrade” branch to generate synthetic LR images, (2) a “learning-to-SR” branch to restore super-resolved images, and (3) another “learning-to-degrade” branch to degrade SR images from real-world LR inputs. Unlike CycleGAN^33^, SCGAN^34^ couples only the “learning-to-SR” branch, ensuring cycle consistency through independent branches.

The lack of paired ground-truth data for real low-resolution faces, together with the substantial domain gap between real and synthetic images, severely limits the ability of existing methods to reconstruct high-resolution face images with realistic appearance. These challenges motivate us to develop a framework capable of producing perceptually convincing and natural face reconstructions directly from real low-resolution inputs. Our method draws inspiration from^35^ and tackles face super-resolution and alignment simultaneously. This is achieved by integrating a sub-network for aligning facial features (FAN)^36^, using heatmap regression, into a Semi-Cycled Generative Adversarial Network (SCGAN)^34^.

The proposed approach harnesses the strengths of SCGAN^34^ to diminish the domain discrepancy between real and synthetic images through dual degradation pathways (forward and backward), operating collaboratively within a cycle-consistency learning framework. To increase the perceptual quality of the synthesized images, a face alignment network (FAN)^36^ is incorporated into the SCGAN^34^ architecture. By aligning facial features via FAN’s heatmap regression and optimizing a dedicated loss, our method preserves fine-grained facial details in super-resolved images. Specifically, FAN predicts 2D landmark heatmaps that provide precise spatial guidance for each facial feature, enforcing structural consistency and ensuring that intricate components–such as eyes, nose, and mouth–are accurately reconstructed. This integration allows the network to produce high-resolution, realistic face images with remarkable precision and goes beyond generic structural information by explicitly maintaining correct geometry and relative positioning of facial features.

Empirical evaluations conducted on real-world as well as synthetic datasets validated the effectiveness of the proposed method compared to existing techniques, achieving exceptional perceptual quality and naturalness. Furthermore, it delivered the highest accuracy in face recognition and detection tasks, effectively preserving critical identity features. This makes the approach particularly suitable for applications that require downstream facial analysis.

The structure of the paper is outlined as follows: Section Related work introduces a review of prior research. Section The proposed approach describes the proposed framework and its architectural design. Section Experiments outlines the experimental setup along with comparisons to state-of-the-art approaches. Section Conclusion concludes the study.

## Related work

### Face super-resolution

The task of human face super-resolution (SR) focuses on generating high-resolution (HR) face images with visually appealing quality from low-resolution (LR) inputs^37^. Earlier approaches to face SR^5–7,38,39^, and^40^ relied heavily on hand-craft image priors and degradation models. For example, Baker et al.^38^ employed Gaussian image pyramids, while Gunturk et al.^39^ proposed a Bayesian framework that addressed SR from a global perspective. To enhance the recovery of local details, methods like those in^5,6^, and^7^ adopted a patch-based modeling strategy, with neighborhood embedding^7^ being a notable example. Subsequent techniques, such as those in^11–14^, and^40^, aimed to balance the reconstruction of local details with the preservation of global structures. Despite their advancements, these methods often struggle to handle complex scenarios in real-world applications.

Deep convolutional neural networks (CNNs) have been leveraged in recent methods^16–19^, and^20^ to address face super-resolution (SR). RBPNet^19^ utilizes iterative back-projection to establish a direct mapping from low-resolution (LR) to high-resolution (HR) face images. SPARNet^18^ incorporates a spatial attention mechanism within its framework to enhance the network’s representational power. WaSRNet^20^ moves the process into the wavelet coefficient domain, enabling better preservation of detailed features. Lu et al.^41^ proposed a combined approach featuring a global upsampling network and a local enhancement network to improve both facial contours and detailed local regions. Because these discriminative learning methods are trained using synthetic images, they often fail to generalize effectively in real-world situations.

Generative models, such as Generative Adversarial Networks (GANs)^24^, have made significant advancements in face super-resolution (SR)^29–32^, and^42^. URDGN^43^ was one of the pioneering methods in this field, but it struggles with low-resolution (LR) face images that exhibit large rotations or varied poses. To address this issue, Super-FAN^35^ employs heatmap regression to pinpoint facial landmarks, enabling it to handle faces with different angles and poses, though it relies on extensive annotated datasets for training. LRGAN^29^ introduces an unsupervised face SR approach using cycle consistency^33^, yet only enforces consistency in high-resolution (HR) images, neglecting consistency in the LR counterparts. PULSE^27^, on the other hand, frequently sacrifices spatial details and identity consistency due to its random sampling of low-dimensional latent codes. More recent methods like GLEAN^44^, GFPGAN^28^, and GPEN^15^ integrate pre-trained StyleGAN^45^ models into their frameworks, but their performance diminishes when dealing with severely degraded LR face images. SCGAN^34^ introduces a novel approach by learning three distinct mappings: two distinct “learning-to-degrade” branches and a shared “learning-to-SR” branch. These mappings are semi-cycled, ensuring consistency in the reconstruction of both high-resolution (HR) and low-resolution (LR) face images.

### Face alignment and landmark localization

Face alignment focuses on localizing predefined facial landmarks and has long been regarded as a fundamental step in face analysis pipelines. The early methods were primarily based on cascaded regression frameworks combined with hand-crafted features, which achieved reasonable performance under controlled conditions but showed limited robustness to pose variation, occlusion, and low-resolution inputs^73^ and^74^.

With the rise of deep learning, convolutional neural network–based approaches significantly improved landmark localization accuracy by learning hierarchical facial representations directly from data^75^. A key breakthrough in this direction was the adoption of heatmap regression, where each landmark is predicted as a spatial probability distribution rather than a single coordinate, enabling better modeling of spatial uncertainty and structural relationships^36^.

Building upon this paradigm, the Face Alignment Network (FAN) introduced stacked hourglass architectures to iteratively refine landmark predictions across multiple scales, achieving state-of-the-art performance on challenging benchmarks^36^ and^66^. Due to its strong geometric modeling capability and robustness, FAN has become a widely adopted baseline for facial landmark localization.

However, standard FAN models are primarily designed for moderate-to-high resolution facial images and often degrade in performance when applied to extremely low-resolution inputs. To address this limitation, SUPER-FAN was proposed to jointly perform face super-resolution and alignment within a unified framework^35^. By coupling super-resolution feature enhancement with landmark heatmap prediction, SUPER-FAN improves alignment reliability under severe resolution constraints. This joint optimization strategy enables structural facial information to be preserved and propagated more effectively, making SUPER-FAN particularly suitable for guiding face super-resolution frameworks.

### Neural network-based image restoration and super-resolution

Deep neural networks have been widely adopted for low-level image restoration tasks, including deblurring, denoising, and super-resolution, due to their strong representation learning capability. Encoder–decoder architectures are among the most commonly used designs, as they effectively capture contextual information while reconstructing spatially coherent structures^67^. Such architectures have been successfully applied to face image enhancement, where preserving both global facial layout and fine local details is critical.

To further improve restoration quality, scale-recurrent and multi-scale networks have been proposed to progressively refine image details across different resolutions^68^. These methods are particularly effective for face image restoration and deblurring, as they allow facial structures to be gradually enhanced while maintaining geometric consistency. By iteratively aggregating information across scales, scale-recurrent designs help alleviate artifacts caused by severe blur or low-resolution degradation, which are common challenges in face super-resolution scenarios.

More recently, advances in modern convolutional network design have demonstrated that carefully optimized CNN architectures remain highly competitive for image restoration and super-resolution tasks. ConvNeXt-based models^69^ revisit classical convolutional principles while incorporating modern training strategies, achieving strong performance without relying on complex attention mechanisms or transformers. These findings highlight that architectural efficiency, multi-scale feature modeling, and effective optimization play a crucial role in high-quality image and face enhancement.

Overall, these neural network–based restoration methods provide important architectural insights for face super-resolution, particularly in terms of multi-scale processing, feature refinement, and structural preservation. Our work builds upon these principles while specifically targeting the unique challenges of face super-resolution, where identity consistency and perceptual realism are of primary importance.

### Cross-domain feature learning and robust representation modeling

Recent studies in infrared-visible object detection and person re-identification, although not directly focused on face super-resolution, provide valuable insights into robust feature modeling. For instance, Deep-IRTarget introduces a dual-domain feature extraction strategy to enhance infrared target detection^70^. Differential Feature Awareness Networks employ antagonistic learning to improve feature allocation under challenging conditions^71^. In addition, visible-infrared person re-identification methods address real-world label noise and domain discrepancies to learn more discriminative representations^72^. These works reflect broader trends toward robust and noise-resilient feature learning, which conceptually align with the structural guidance mechanisms adopted in our framework to stabilize super-resolution performance.

Building upon the advancements of SCGAN^34^ in constructing high-resolution facial images from low-quality real-world ones, and drawing inspiration from Super-FAN’s integration of a face alignment sub-network^35^ into a GAN-based super-resolution framework, in this study we specifically incorporate a face alignment sub-network^36^, utilizing heatmap regression, into the SCGAN architecture^34^. This sub-network is integrated into the forward cycle, which is responsible for synthesizing high-resolution facial images, thereby enhancing the alignment between real and synthesized images by minimizing the heatmap loss. These heatmaps encode essential geometric priors that guide the reconstruction process and help maintain facial consistency throughout the super-resolution semi-cycled. Through this approach, we present an unsupervised face super-resolution framework that demonstrates the capability to generate high-resolution facial images with remarkable perceptual quality and natural realism.

## The proposed approach

**Fig. 1:**
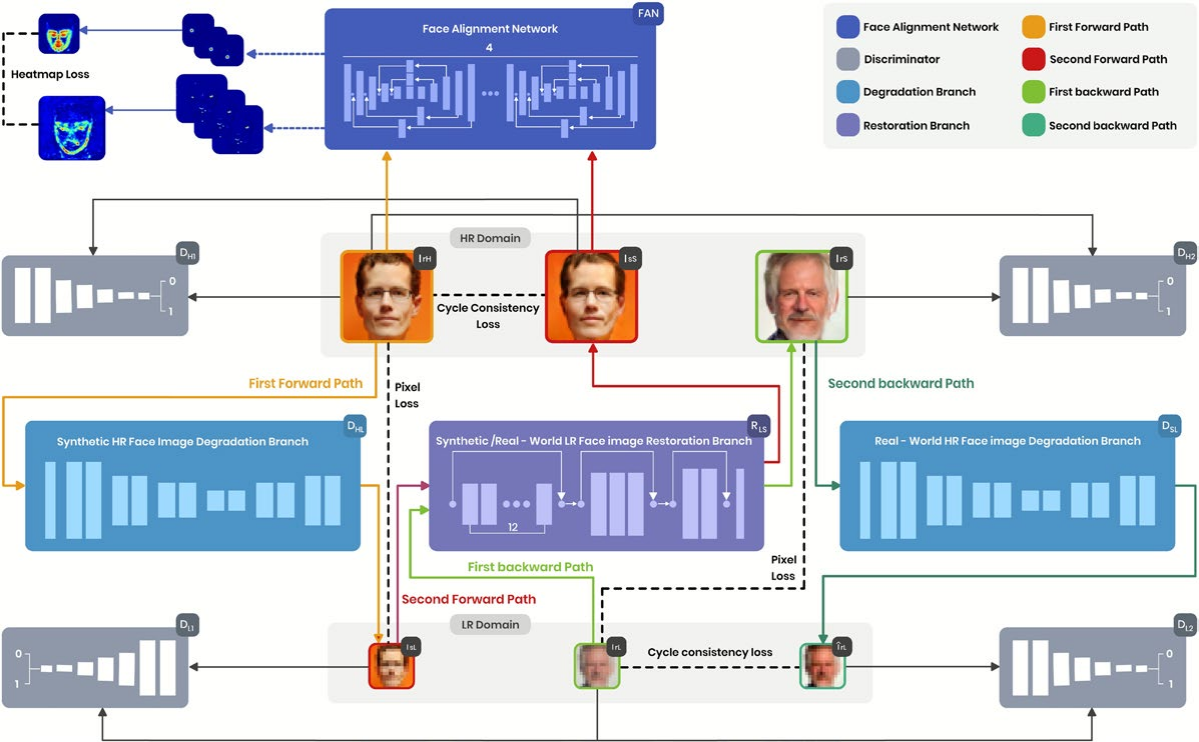
Architecture of our proposed framework that incorporates a face alignment network (FAN)^36^ within a semi-cycle generative adversarial network (SCGAN)^34^, for enabling enhanced unsupervised face image super-resolution (SR). Our architecture comprises forward and backward semi-cycled sub-networks. The forward semi-cycle is carried out through the first forward path (represented by the orange-colored lines), the second forward path (represented by the red-colored lines) and then the face alignment network (FAN). The first forward path represents the path for a real-world high-resolution face image to be degraded to its synthetic low-resolution version through synthetic HR face image degradation branch $D_{HL}$, which in turn goes through the second forward path for restoring its super-resolved version through synthetic LR face image restoration branch $R_{LS}$. Last step in the forward semi-cycle is accomplished by passing the real-world high-resolution and the synthetic super-resolved face images to the face alignment network to improve the alignment between real and super-resolved images. The backward semi-cycle is represented by the green lines and carried out through the first backward path (represented by the light green lines) and the second backward path (represented by the dark green lines). The first backward path represents the path for a real-world low-resolution face image to be super-resolved through real-world LR face image restoration branch $R_{LS}$, which in turn goes through the second backward path to be degraded to its synthetic low-resolution version via the real-world HR face image degradation branch $D_{SL}$.

This section elaborates on our approach for constructing high-resolution face images with outstanding perceptual quality and naturalness. The proposed architecture, illustrated in Fig. 1, incorporates a face alignment network (FAN)^36^ within a semi-cycle generative adversarial network^34^, designed for unsupervised face image super-resolution (SR). Our architecture comprises forward and backward semi-cycled sub-networks, each featuring an independent degradation branch interconnected through a shared restoration branch. The forward semi-cycled sub-network, detailed in The forward semi-cycled sub-network subsection and illustrated in Fig. 1 using orange and red lines, includes a synthetic HR image degradation branch, a synthetic LR face restoration branch, together with a face alignment network, which collectively conduct forward cycle-consistent reconstruction of HR face images. The backward semi-cycled sub-network, explained in The backward semi-cycled sub-network subsection and illustrated in Fig. 1 using light and dark green lines, comprises a real-world LR face restoration branch and a real-world HR image degradation branch, which work together to achieve backward cycle-consistent reconstruction of LR face images. Notably, the synthetic/real-world LR face restoration branch serves as a shared hub for the forward and backward cycle-consistency learning processes. The two reconstruction sub-networks are designed to operate in a semi-cycled manner, effectively mitigating the adverse effects arising from the domain discrepancy between synthetic and real-world LR face images. This approach ensures a seamless adaptation between the two domains, enabling the framework to achieve robust and precise face super-resolution (SR) performance while maintaining high perceptual quality and consistency. Finally, the loss functions related to the forward and backward semi-cycled sub-networks explained in details in Loss functions subsection.As our proposed approach based mainly on integrating a face alignment network (FAN)^36^ into a semi-cycle generative adversarial network (SCGAN)^34^, We have used SCGAN’s^34^ abbreviations and equations except for the equations that were affected by our integration of FAN.

### The forward semi-cycled sub-network

This sub-network is specifically designed to learn the degradation kernel that transforms real-world high-resolution (HR) face images into synthetic low-resolution (LR) face images and to refine the degraded synthetic image by preserving essential details and suppressing artifacts generated throughout the degradation procedure, ensuring a more accurate and realistic super-resolved face images. It composed of three essential components: a synthetic HR image degradation branch, a synthetic LR face restoration branch, and a face alignment network. The degradation branch simulates the process of converting HR images into LR ones, effectively modeling real-world image degradation. The restoration branch is concerned with reconstructing HR images from the degraded LR inputs, emphasizing the preservation of fine details and facial features. Meanwhile, the face alignment network ensures geometric consistency by maintaining key facial structures, such as landmarks, throughout the reconstruction process.

Together, these components work in harmony to achieve forward cycle-consistent reconstruction of HR face images. The degradation and restoration branches handle the transformation between resolutions, while the face alignment network ensures structural accuracy and alignment. This integrated design not only facilitates the generation of visually appealing HR face images but also ensures consistency, bridging the gap between real-world and synthetic data. By doing so, the forward semi-cycled sub-network offers a robust solution for face super-resolution tasks, producing high-quality results that are both perceptually natural and structurally coherent. In the subsequent section, we present an in-depth explanation of the sub-network components and then highlight the forward semi-cycled losses.

#### Synthetic HR face image degradation branch $D_{HL}$

The goal of this branch is to artificially degrade high-resolution (HR) real-world facial images into their corresponding synthetic LR versions. The synthetic HR image degradation branch replicates the process of transforming HR images into LR ones, effectively capturing the image degradation that occurs in real-world scenarios. Following the approach outlined in^46^, to mimic various levels and types of noise commonly found in real-world LR face images, a noise vector $z\in R^{HW}$ is randomly generated, reshaped to dimensions $H\times W$, and concatenated with the HR face image $I_{rH} \in R^{H\times W \times 3}$ along the channel dimension.

This results in a concatenated tensor $[I_{rH},z]\in R^{H\times W \times 4}$, which is then processed through the degradation branch $D_{HL}$. The degradation branch applies transformations to simulate downscaling and noise addition, generating a synthetic LR face image $I_{sL}$. This process not only simulates the visual effects of degradation, but also provides a robust training setup by introducing noise variability, ensuring that the model is capable of handling a wide range of real-world LR image conditions.

1 $$\begin{aligned} I_{sL} = D_{HL} ([I_{rH},z],\theta _{HL}) \end{aligned}$$

with,$\theta _{HL}$ serving as the set of learnable parameters for the $D_{HL}$model.

**Fig. 2:**
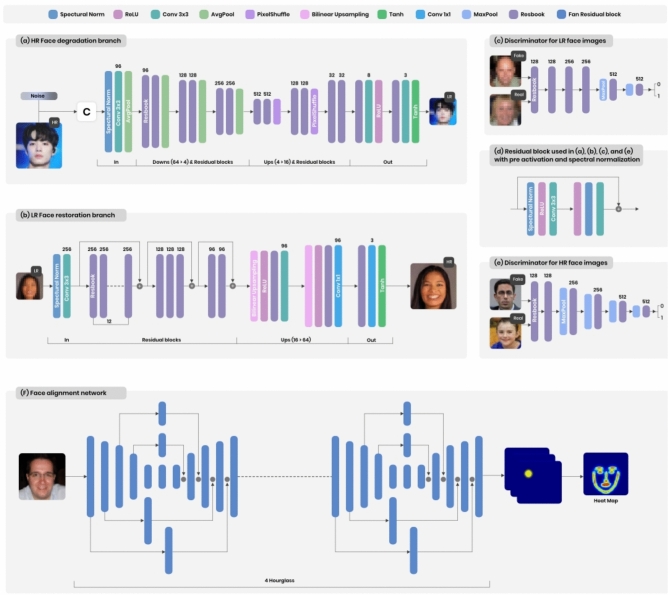
Structural layouts of the synthetic and real-world HR face image degradation branches $D_{HL}$ and $D_{SL}$ (**a**), the LR face image restoration branch $R_{LS}$ (**b**). The discriminators networks are depicted in (**c**) and (**e**), while the residual block employed within them is illustrated in (**d**). The Face Alignment Network is shown in (**f**).

The synthetic HR image degradation branch, illustrated in Fig. 2.a, utilizes an encoder-decoder architecture, as in^34^, to effectively simulate the process of degrading HR images into synthetic LR counterparts. The encoder starts by incorporating Spectral Normalization (SN)^47^, which is followed by a $3 \times 3$ convolutional layer and global average pooling (GAP). After these initial layers, six residual blocks (Residual Blocks) are employed for feature extraction from the input. Each ResBlock, as depicted in Fig. 2.d, comprises two sequences of SN, ReLU activations, $3 \times 3$ convolutions, and a skip connection. The application of SN ensures stable training by enforcing the 1-Lipschitz constraint, effectively preventing issues such as gradient explosion^47^. GAP further reduces the resolution of the feature maps by half after every two Residual Blocks, enhancing the efficiency of feature processing.

The decoder mirrors the architecture of the encoder, consisting of six Residual Blocks and incorporating Pixel-Shuffle operations after the second and fourth Residual Blocks to upsample the resolution. It concludes with two additional Residual Blocks, $3 \times 3$ convolutions, and an activation layer (either ReLU or Tanh) to generate the final degraded LR face image. By leveraging this encoder-decoder framework, the branch effectively models the degradation process, producing synthetic LR images $I_{sL}$ that realistically simulate real-world conditions while maintaining key structural and textural elements.

#### Synthetic/real-world LR face image restoration branch $R_{LS}$

The low-resolution (LR) face restoration branch $R_{LS}$, as proposed in^34^, serves as a pivotal component that bridges forward and backward cycle-consistency learning processes. The branch incorporates sophisticated feature extraction mechanisms, leveraging deep convolutional layers and advanced loss functions to enhance the accuracy of restorations. It plays a dual role in ensuring the accuracy and consistency of the reconstructed face images across both learning cycles. In the forward learning process, the branch is responsible for restoring the synthetic LR face image $I_{SL}$, which is produced by degrading the high-resolution (HR) face image $I_{rH}$, using the degradation branch $D_{HL}$. This process ensures that the synthetic LR image can be super-resolved to closely resemble its original HR counterpart. Meanwhile, in the backward learning process, $R_{LS}$ takes real-world LR face images $I_{rL}$, as input, and restores them into super-resolved (SR) images that retain essential details and visual fidelity. This dual functionality allows the branch to generalize effectively across synthetic and real-world scenarios, improving the overall robustness and adaptability of the framework. By integrating the restoration processes for both synthetic and real-world LR images within a shared framework, $R_{LS}$ ensures that cycle-consistency learning remains coherent and efficient. This design not only enforces alignment between forward and backward processes, but also significantly contributes to the framework’s ability to handle diverse types of LR inputs with consistent and high-quality outputs. The restoration mechanism therefore acts as a cornerstone in achieving realistic and perceptually compelling SR results. The detailed restoration steps in the forward learning process are as follows:

2 $$\begin{aligned} I_{sS} = R_{LS}(I_{sL},\theta _{LS}) \end{aligned}$$

where, $I_{sS}$ symbolize the SR image reconstructed from $I_{sL}$, and $\theta _{LS}$ denotes the set of learnable parameters for the $R_{LS}$ model.

Figure 2b depicts that the restoration branch $R_{LS}$ employs a robust architecture designed to effectively reconstruct high-resolution (HR) face images. The branch begins with Spectral Normalization (SN)^47^ to stabilize training and ensure consistent feature extraction, followed by a $3 \times 3$ convolutional layer for the initial processing of input features. To extract meaningful features, the architecture incorporates three groups of Residual Blocks containing 12, 3, and 2 blocks, respectively. These Residual Blocks serve a pivotal function in refining the features and preserving important image details. Each group is equipped with skip connections that link the input and output, facilitating feature addition and retaining high-frequency details crucial for sharp image reconstruction. To enhance the resolution of the feature map, the branch applies two bilinear interpolation steps, each upscaling the resolution by a factor of 2, resulting in an overall upsampling by a factor of 4. After upsampling, the architecture processes the feature map through two groups of layers: the first group comprises a “ReLU-ResBlock-$3 \times 3$ Conv.,” and the second group includes a “ReLU-ResBlock-$1 \times 1$ Conv.” These groups further refine the features and prepare them for final reconstruction. The restoration branch concludes with a Residual Block, followed by a $1 \times 1$ convolutional layer and a Tanh activation function to output the restored HR face image. This carefully designed pipeline ensures that the branch can reconstruct images with exceptional clarity and fidelity, effectively alleviating the domain discrepancy between low-resolution inputs and high-resolution outputs.

#### Face alignment network *FAN*

**Fig. 3:**
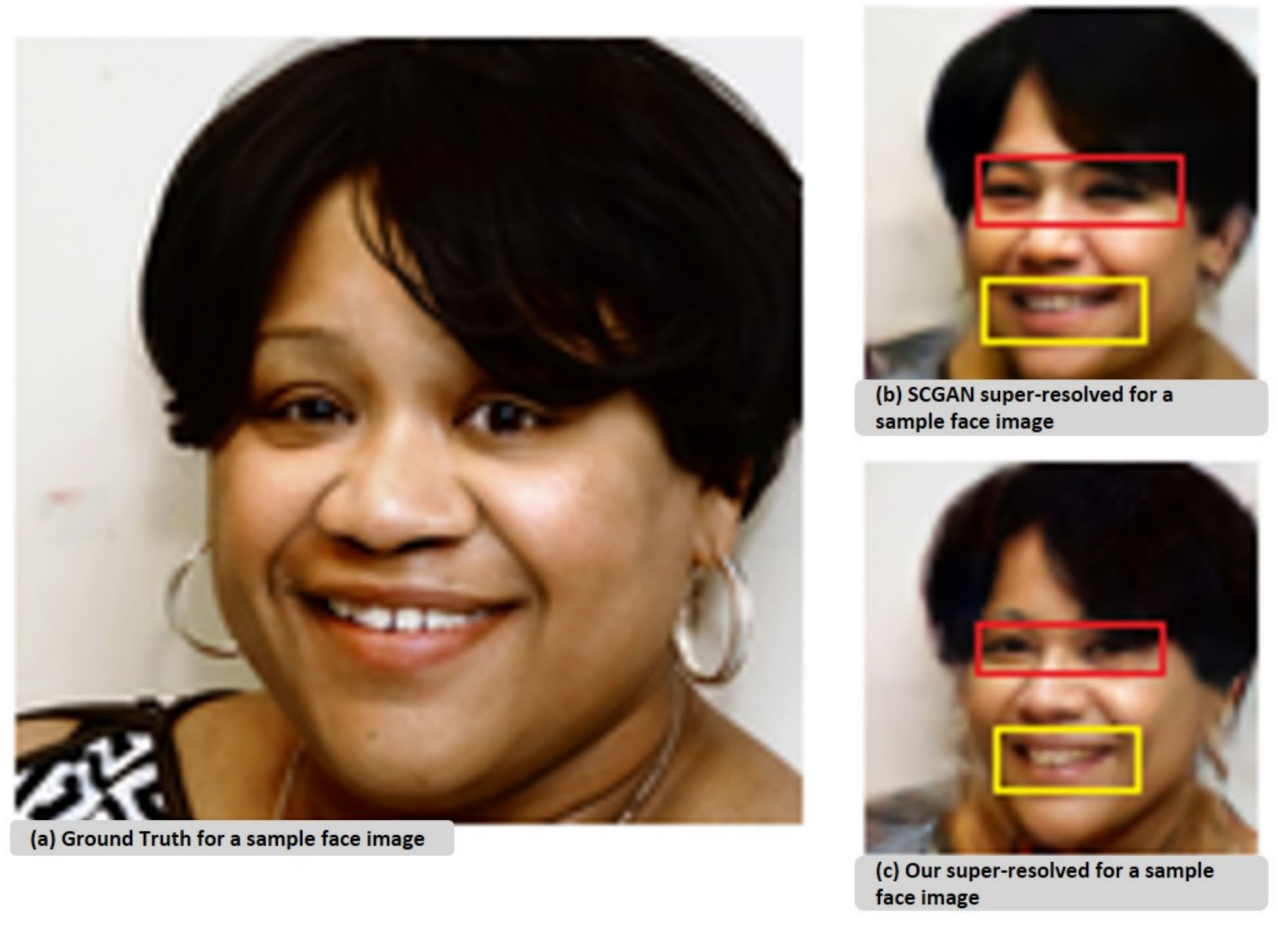
Sample face image from FFHQ^45^ to show the effect of incorporating structural information into super-resolution process, (**a**) The ground truth face image, (**b**) SCGAN super-resolved face image, (**c**) our super-resolved face image. The figure shows a missing details in eyes and lips in the sample which doesn’t incorporate structural information as outlined using red and yellow rectangles. Please zoom in for the best view.

Through our observations, we found that relying solely on the previously defined losses (pixel and adversarial) can lead to missing details related to pose or facial expressions or misalignment of facial features. This limitation arises because these losses do not incorporate structural information about the human face into the super-resolution process, as illustrated in Figure 3.

To address this issue, we draw inspiration from^35^ and introduce a facial landmark localization sub-network FAN^36^ into the super-resolution framework SCGAN^34^. By leveraging heatmap regression, this sub-network enforces structural consistency between low-resolution and high-resolution face images. Additionally, we optimize a dedicated heatmap loss to further enhance the preservation of facial features throughout the super-resolution process.

To achieve this, we integrate the face alignment sub-network FAN^36^ into the forward semi-cycled sub-network. After the synthetic low-resolution face image $I_{sL}$ is restored using the $R_{LS}$ branch, the resulting synthetic super-resolved face image $I_{sS}$ is fed into FAN. The network is then trained to ensure that its output matches the output of another FAN applied to the real-world high-resolution face image $I_{rH}$. This approach reinforces structural consistency and improves facial alignment in the super-resolution process.

Regarding the architectural design, we employed FAN^36^ which particularly uses four Hourglass modules, as illustrated in Fig. 2f. This structure effectively captures multi-scale facial features, enhancing the accuracy of facial landmark localization within the super-resolution process.

FAN^36^ utilizes heatmap regression to accurately localize facial landmarks. Rather than directly regressing a $68 \times 2$vector of x and y coordinates, each facial landmark is encoded as an output channel containing a 2D Gaussian distribution centered at its location. The network is then trained for regression of these 2D Gaussians, commonly referred to as heatmaps. Previous studies, for example^48^, have demonstrated that these heatmaps encapsulate crucial facial attributes such as pose, expression, spatial context, and structural relationships between different facial parts. A fundamental aspect of our approach is enforcing similarity between the heatmaps generated from the synthetic super-resolved face image $I_{sS}$ and those from the corresponding real-world high-resolution face image $I_{rH}$. By minimizing the discrepancy between these heatmaps, we not only ensure precise landmark localization but also impose structural consistency between the synthetic super-resolved and real-world high-resolution face images.

### The backward semi-cycled sub-network

This sub-network is specifically designed to learn the degradation kernel that transforms real-world high-resolution (HR) face images into synthetic low-resolution (LR) face images and to refine the degraded synthetic image by preserving essential details and reducing artifacts introduced during the degradation.

The backward and forward sub-networks are interconnected in a semi-cycled manner to mitigate the negative impact of the domain discrepancy between synthetic and real-world low-resolution (LR) face images. This design ensures seamless adaptation across domains, enabling the framework to deliver robust and precise face super-resolution (SR) performance while maintaining high fidelity and consistency in image reconstruction. Therefore, the backward sub-network is designed to degrade real-world super-resolved (SR) face images into synthetic low-resolution face images that exhibit high fidelity to real-world low-resolution counterparts. It consists of two key components: Real-world LR face restoration branch and Real-world HR image degradation branch.

#### Synthetic/real-world LR face image restoration branch $R_{LS}$

The synthetic/real-world LR face restoration branch $R_{LS}$ serves as the central hub shared by both the forward and backward cycle-consistency learning processes. Its functionality and detailed design are thoroughly discussed in Synthetic/Real-world LR face image restoration branch $R_{LS}$ subsection and Figure 2b, highlighting its critical role in ensuring effective and consistent learning across both cycles. As mentioned in Synthetic/Real-world LR face image restoration branch $R_{LS}$ subsection, in the backward learning process, $R_{LS}$ accepts real-world LR face images $I_{rL}$ as input and restores them into super-resolved (SR) images that preserve essential details and visual fidelity. Therefore, we can represent the restoration steps in the backward learning process are as follows:

3 $$\begin{aligned} I_{rS} = R_{LS}(I_{rL},\theta _{LS}) \end{aligned}$$

with, $I_{rS}$ serving as the SR image reconstructed from $I_{rL}$, and $\theta _{LS}$ denotes the set of learnable parameters for the $R_{LS}$ model.

#### Real-world HR face image degradation branch $D_{SL}$

This branch is responsible for learning the degradation process applied to super-resolved (SR) face images $I_{rS}$, which have been restored from the real-world low-resolution (LR) face image $I_{rL}$ with the aim of producing degradation results that closely resemble real-world low-resolution (LR) face images. The architecture of $D_{SL}$ mirrors that of the Synthetic HR face image degradation branch $D_{HL}$, as introduced in Synthetic HR face image degradation branch $D_{HL}$ subsection and Fig. 2a. By simulating this degradation, the branch ensures consistency between the reconstructed and degraded images, facilitating effective backward cycle-consistent learning. Therefore, the degradation process of $D_{SL}$ can be represented as follows:

4 $$\begin{aligned} \hat{I}_{rL} = D_{SL}(I_{rS},\theta _{SL}) \end{aligned}$$

with, $\hat{I}_{rL}$ the LR degradation output, and $\theta _{SL}$ denotes the set of learnable parameters for the $D_{SL}$ model.

### Loss functions

This section offers a comprehensive description of the loss functions used for the three preceding branches.

#### Loss functions for $D_{HL}$ branch

To closely mimic the degradation kernel present in real-world low-resolution (LR) face images, the synthetic HR face image degradation branch $D_{HL}$ is trained using the following loss functions:

- **Pixel loss:** In order to effectively reconstruct image details, the pixel loss $l_{pix}^{I_{sL}}$ is computed between the synthetic degraded image $I_{sL}$ and the input HR face image $I_{rH}$, which is downsampled to match the resolution of $I_{sL}$ using average pooling. For this calculation, we utilize the $\ell _1$ loss function which is commonly employed in image super-resolution tasks^29^ and^49^.
- **Adversarial loss:** With the purpose of differentiating between real-world LR face images $I_{rL}$ and synthetic LR images $I_{sL}$, the adversarial loss $l_{adv}^{D_{L1}}$ employs a discriminator $D_{L1}$, which assigns a label of 1 for $I_{rL}$ and a label of 0 for $I_{sL}$. As illustrated in Fig. 2c, $D_{L1}$ consists of six Residual Blocks followed by a fully connected layer. To reduce the feature map resolution, max-pooling is applied before the final two Residual Blocks. The adversarial loss $l_{adv}^{D_{L1}}$ is formulated in the following manner:
  5 $$\begin{aligned} l_{adv}^{D_{L1}} = \mathbb {E}_{I_{rL} \sim P_{rL}} \left[ \min \left( 0, D_{L1}(I_{rL}) - 1\right) \right] + \mathbb {E}_{I_{sL} \sim P_{sL}} \left[ \min \left( 0, -1 - D_{L1}(I_{sL})\right) \right] \end{aligned}$$
  Here, $P_{rL}$ and $P_{sL}$, represent the distributions of the real-world LR face image $I_{rL}$ and the synthetic LR face image $I_{sL}$, which is obtained by degrading the real-world high-resolution (HR) face image $I_{rH}$, through $D_{HL}$.

Therefore, the total loss for the synthetic HR face image degradation branch $D_{HL}$ is:

6 $$\begin{aligned} l_{D_{HL}} = \alpha \, l_{adv}^{D_{L1}} + \beta \, l_{pix}^{I_{sL}} \end{aligned}$$

with $\alpha$ and $\beta$ serving as the weighting factors assigned to the loss functions.

#### Loss functions for $R_{LS}$ branch

To construct high-quality super-resolved (SR) face images, the restoration branch $R_{LS}$ focuses on refining the outputs by preserving intricate details and minimizing artifacts. This branch is a shared component of both the forward and backward learning processes, ensuring consistency and robustness in image restoration. Training $R_{LS}$ involves optimizing a combination of loss functions tailored to each learning process. In the forward learning process, the training objective incorporates the following three loss functions to enhance realism, facial alignment, and structural coherence:

- **Adversarial loss:** A discriminator $D_{H1}$ is employed to distinguish between real-world high-resolution (HR) face images $I_{rH}$ and their synthetic super-resolved counterparts $I_{sS}$, assigning a label of 1 to real HR images and 0 to synthetic ones. The discriminator $D_{H1}$ is structured with six Residual Blocks followed by a fully connected layer. To enhance feature extraction and reduce spatial dimensions, max-pooling is applied before the last four Residual Blocks. This architectural design, as illustrated in Fig. 2e, enables this adversarial learning mechanism enhances the realism of generated images by encouraging the model to produce super-resolved faces that closely resemble real-world data. The adversarial loss $l_{adv}^{D_{H1}}$ is calculated in the following manner:
  7 $$\begin{aligned} l_{adv}^{D_{H1}} = \mathbb {E}_{I_{rH} \sim P_{rH}} \left[ \min \left( 0, D_{H1}(I_{rH}) - 1\right) \right] + \mathbb {E}_{I_{sS} \sim P_{sS}} \left[ \min \left( 0, -1 - D_{H1}(I_{sS})\right) \right] \end{aligned}$$
  where, $P_{rH}$ and $P_{sS}$,denote the distributions of the real-world high-resolution (HR) face image $I_{rH }$ and the synthetic super-resolved image $I_{sS}$,respectively. The latter is reconstructed from the real-world low-resolution face image $I_{sL}$ using $R_{LS}$ branch.
- **Heatmap loss:** To maintain structural consistency, we enforce alignment between the real-world high-resolution face image $I_{rH}$ and their corresponding synthetic super-resolved face image $I_{sS}$ using a heatmap loss function $l_{heatmap}^{I_{sS}}$, ensuring that facial features remain spatially accurate and well-preserved. The heatmap loss $l_{heatmap}^{I_{sS}}$ is formulated as follows:
  8 $$\begin{aligned} l_{heatmap}^{I_{sS}} = \frac{1}{N} \sum _{n=1}^{N} \sum _{i,j} \left( \tilde{M}_{i,j}^{n} - \hat{M}_{i,j}^{n} \right) ^2 \end{aligned}$$
  Here, *N* represents the landmarks’ number, $\hat{M}_{i,j}^{n}$ and $\tilde{M}_{i,j}^{n}$ represent the heatmaps associated with the n-th landmark at pixel (i, j), generated from running a FAN on the real-world high-resolution face image $I_{rH}$ and their corresponding synthetic super-resolved face image $I_{sS}$, respectively. A significant advantage that encourages us to use the heatmap loss is that it eliminates the need for ground truth landmark annotations, relying solely on a pre-trained FAN^36^. This enables the training of the complete super-resolution framework under a weakly supervised manner.
- **Cycle-consistency loss:** For ensuring that the restoration branch $R_{LS}$ effectively preserves identity information and accurately reconstructs facial details, we used a cycle-consistency loss $l_{cyc}^{I_{sS}}$, which is implemented as an $\ell _1$ loss function. This loss encourages structural consistency and high-fidelity face recovery, leading to more realistic and visually coherent super-resolved outputs by minimizing the discrepancy between the original and restored images.

Therefore, the total loss function for the forward learning process is represented as:

9 $$\begin{aligned} l_{R_{LS}}^{I_{sS}} = \alpha \, l_{adv}^{D_{H1}} + \beta \, l_{cyc}^{I_{sS}} + \lambda \, l_{heatmap}^{I_{sS}} \end{aligned}$$

with $\alpha$, $\beta$ and $\lambda$ serving as weighting factors assigned to loss functions.

Meanwhile, in the backward learning process, a combination of adversarial loss and pixel loss is used to refine the reconstructed images, ensuring fidelity to real-world low-resolution face images while maintaining high perceptual quality:

- **Adversarial loss:** A discriminator $D_{H2}$ is utilized to differentiate between real-world high-resolution (HR) face images $I_{rH}$ and real-world super-resolved (SR) images $I_{rS}$, assigning a label of 1 to HR images and 0 to SR images. The discriminator $D_{H2}$ has the same structure as $D_{H1}$ which is outlined in Fig. 2e. This adversarial framework encourages the model to generate SR images that exhibit high fidelity to real HR images, enhancing their realism and perceptual quality. The adversarial loss $l_{adv}^{D_{H2}}$, is calculated in the following manner:
  10 $$\begin{aligned} l_{adv}^{D_{H2}} = \mathbb {E}_{I_{rH} \sim P_{rH}} \left[ \min \left( 0, D_{H2}(I_{rH}) - 1\right) \right] + \mathbb {E}_{I_{rS} \sim P_{rS}} \left[ \min \left( 0, -1 - D_{H2}(I_{rS})\right) \right] \end{aligned}$$
  where,$P_{rH}$ and $P_{rS}$, denote the distributions of the real-world high-resolution (HR) face image $I_{rH}$ and the real-world super-resolved image $I_{rS}$, respectively. The latter is reconstructed from the real-world low-resolution face image $I_{rL}$ using the $R_{LS}$ branch.
- **Pixel loss:** In order to penalize the difference between the real-world low-resolution (LR) face images and their super-resolved (SR) counterparts $I_{rS}$, first we upsampled the real-world low-resolution face image $I_{rL}$ to match the same size as $I_{rS}$ using bicubic interpolation. We employed a pixel loss function $l_{pix}^{I_{rS}}$, defined as an $\ell _1$ loss function. By minimizing this loss, it ensures that the SR image closely resembles the original HR image in terms of structure and detail.

Therefore, the total loss function for the backward learning process is represented as:

11 $$\begin{aligned} l_{R_{LS}}^{I_{rS}} = \alpha \, l_{adv}^{D_{H2}} + \beta \, l_{pix}^{I_{rS}} \end{aligned}$$

with $\alpha$, and $\beta$ serving as the weighting factors assigned to the loss functions.

Concluding, the total loss for the Synthetic/Real-World LR face Restoration branch $R_{LS}$ is:

12 $$\begin{aligned} l_{R_{LS}}= \theta \, l_{R_{LS}}^{I_{sS}} + \gamma \, l_{R_{LS}}^{I_{rS}} \end{aligned}$$

with $\theta$, and $\gamma$ serving as the weighting factors assigned to the loss functions.

#### Loss functions for $D_{SL}$ branch

To closely resemble the degradation kernel present in real-world low-resolution (LR) face images, the real-world HR image degradation branch $D_{SL}$ is trained using the following loss functions:

- **Adversarial loss:** To differentiate between real-world low-resolution (LR) face images $I_{rL}$ and the degraded version $\hat{I}_{rL}$ of real-world super-resolved images $I_{rS}$ using $D_{SL}$, a discriminator $D_{L2}$ is employed. It assigns a label of 1 to $I_{rL}$ and 0 to $\hat{I}_{rL}$, ensuring that the degradation process closely mimics real-world conditions. The structure of $D_{L2}$ is identical to that of $D_{L1}$, as detailed in Loss functions for $D_{HL}$ branch subsection and depicted in Fig. 2c. The adversarial loss $l_{adv}^{D_{L2}}$ is calculated in the following manner:
  13 $$\begin{aligned} l_{adv}^{D_{L2}} = \mathbb {E}_{I_{rL} \sim P_{rL}} \left[ \min \left( 0, D_{L2}(I_{rL}) - 1\right) \right] + \mathbb {E}_{\hat{I}_{rL} \sim P_{\hat{rL}}} \left[ \min \left( 0, -1 - D_{L2}(\hat{I}_{rL})\right) \right] \end{aligned}$$
  Here, $P_{rL}$ and$P_{\hat{rL}}$, represent the distributions of the real-world low-resolution (LR) face image $I_{rL}$ and the the degraded version $\hat{I}_{rL}$ of real-world super-resolved images $I_{rS}$ using $D_{SL}$.

- **Cycle-consistency loss:** To mitigate the discrepancy between the degraded low-resolution (LR) image $\hat{I}_{rL}$, produced by this branch, and its corresponding real-world LR face image $I_{rL}$, a cycle-consistency loss $l_{cyc}^{\hat{I}_{rL}}$ is utilized, which is implemented as an $\ell _1$ loss function. By enforcing this loss, the model is guided to generate degraded images that closely resemble actual LR face images, ensuring a more realistic and accurate degradation process.

Therefore, the total loss Real-World HR image degradation branch $D_{SL}$is:

14 $$\begin{aligned} l_{D_{SL}} = \alpha \, l_{adv}^{D_{L2}} + \beta \, l_{cyc}^{\hat{I_{rL}}} \end{aligned}$$

with $\alpha$, and $\beta$ serving as the weighting factors assigned to the loss functions.

## Experiments

This section begins by outlining the experimental setup, dataset, and evaluation metrics in Experimental setup subsection. Through Comparisons with top-performing approaches subsection, we present a comparison alongside top-performing approaches for real-world face super-resolution (SR). Lastly, in Assessment on downstream vision tasks subsection, we demonstrate the applicability of our approach to related vision tasks, including face detection and face verification.

### Experimental setup

Following the exact implementation specifications in the existing work, SCGAN^34^, the parameters for all three branches in our framework are initialized using Kaiming initialization^50^, and optimized with the Adam optimizer^51^, setting $\beta \ 1 = 0.9$ and $\beta \ 2 = 0.999$. For loss functions, we set $\alpha \ = 1$ and $\beta \ = 0.05$ in Eqs. 6, 9, 11, and 14, $\lambda \ = 1$ in Eq. 9, and $\theta \ = 1$ and $\gamma \ = 0.05$ in Eq. 12. Our framework is trained for 200 epochs. The learning rate starts at $1 \times 10^{-4}$ and is decayed to $1 \times 10^{-5}$ using the cosineannealing scheme every 10 epochs. For generating the heatmap loss, we utilized a pre-trained FAN^36^, following the implementation details in^36^.The batch size is configured to 64. We implement our framework using PyTorch^52^ and train it on a NVIDIA TESLA P100 GPUs provided by kaggle^53^, requiring approximately 100 hours for the entire training process. Table 1 shows the average training time per epoch on a single NVIDIA Tesla P100 GPU. Our model requires about 30 minutes per epoch, comparable to SCGAN^34^, demonstrating that the FAN-based structural guidance does not add significant computational overhead.

**Table 1:** Average training time per epoch on a single NVIDIA Tesla P100 GPU.

Method	Average Training Time per Epoch (min)
SCGAN^34^	10.17
Ours	30.09

#### Training/testing sets

Following the methodology established in SCGAN^34^, the training dataset consists of 20,000 high-resolution (HR) face images sourced from the FFHQ dataset^45^, which provides high-quality and diverse facial images. Additionally, we incorporate 4,000 low-resolution (LR) face images obtained from the real-world Widerface dataset^54^, ensuring that our model learns to handle real-world low-quality facial images effectively. This combination allows our framework to generalize well across different levels of image quality and diverse facial structures.

For testing purposes, we assess the performance of our method and comparison approaches using four widely recognized face super-resolution (SR) datasets. These include two synthetic datasets–LS3D-W Balanced^36^ and FFHQ^45^–as well as two real-world datasets–Widerface^54^ and Webface^34^. The synthetic datasets provide high-quality paired data, facilitating controlled experiments, while the real-world datasets introduce challenges such as noise, compression artifacts, and diverse lighting conditions, ensuring a comprehensive evaluation of our model’s effectiveness in practical scenarios.

All aforementioned datasets are preprocessed following the same procedure as SCGAN^34^, to ensure consistency in evaluation. For all experiments, we apply a 4$\times$ super-resolution factor, meaning that the low-resolution images are upscaled by a factor of four to generate high-resolution counterparts. This standardized processing allows for a fair comparison across.

All datasets employed in this study are publicly available and were originally released with the necessary ethical approvals and informed consent.

#### Evaluation metrics

To objectively and comprehensively assess the performance of different super-resolution methods, we utilize both feature-level and image-level evaluation metrics. Across all test datasets, we compute the Fréchet Inception Distance (FID)^55^ to measure the similarity between the distribution of the generated super-resolved (SR) images and real-world high-resolution (HR) face image (for our testing we used the same 20000 (HR) face images sourced from the FFHQ dataset^45^), which reflects diversity and overall visual fidelity. Additionally, the Kernel Inception Distance (KID)^56^ is employed to further evaluate visual quality. For synthetic datasets, we employ the Learned Perceptual Image Patch Similarity (LPIPS)^57^ metric to measure the perceptual distance between the super-resolved (SR) images and their corresponding ground-truth high-resolution (HR) counterparts that align closely with human visual judgments, making it particularly suitable for evaluating the visual quality of restored images. In addition, we incorporate pixel-based evaluation metrics, namely PSNR^76^ and SSIM^77^, to complement the perceptual assessment. PSNR quantifies the pixel-wise reconstruction fidelity between the SR images and the ground-truth HR images, while SSIM evaluates structural similarity by considering luminance, contrast, and structural information. In contrast, for real-world datasets, where ground-truth HR images are unavailable, we use the widely adopted Natural Image Quality Evaluator (NIQE)^58^ to assess the realism and naturalness of the restored face images. Beyond visual quality assessments, we evaluate identity preservation by computing face detection accuracy using the Histogram of Oriented Gradients (HOG) combined with a Support Vector Machine (SVM) classifier^59^. This metric provides an indirect measure of how well the SR methods retain crucial facial features that contribute to accurate face recognition and verification.

#### Parameter sensitivity analysis

To systematically assess the influence of loss weighting on reconstruction performance, we conduct a focused sensitivity analysis on the coefficient $\lambda$, which regulates the contribution of the proposed FAN-guided structural loss. The remaining loss weights $\alpha$,$\beta$,$\theta$, and $\gamma$ are fixed to the values adopted in SCGAN^34^, as they correspond to established loss components whose stability and effectiveness have been previously validated. This controlled setup allows us to isolate the effect of the newly introduced structural constraint and avoid confounding interactions among multiple hyperparameters. Quantitative results on the FFHQ dataset, summarized in Table 2 using FID, KID, and LPIPS metrics, reveal how varying $\lambda$ impacts perceptual quality and distributional alignment, thereby providing direct insight into the contribution of the proposed structural guidance. The results show that $\lambda \ = 1$ consistently yields the best perceptual performance, achieving the lowest FID, KID, and LPIPS scores. This study indicates that the proposed method is not highly sensitive to parameter variation and performs robustly under a reasonable range of $\lambda$ values.

**Table 2:** Parameter sensitivity analysis of the loss weight $\lambda$ on the FFHQ^45^dataset. The remaining loss weights are fixed following SCGAN^34^. Best results are highlighted in italic.

$\lambda$	FID$\downarrow$	KID$\downarrow$	LPIPS$\downarrow$
0.0005	25.31	$1.7\pm 0.06$	0.1988
0.5	24.16	$1.5\pm 0.07$	0.1976
0.9	23.18	$1.4\pm 0.06$	0.1952
1	*22.48*	*1.26*$\pm$*0.06*	*0.1896*

### Comparisons with top-performing approaches

We perform both quantitative and qualitative comparisons with Bicubic Interpolation and state-of-the-art methods, including HiFaceGAN^60^, Real-ESRGAN^61^, GFPGAN^28^, LRGAN^29^, and PULSE^27^. Our evaluation uses two synthetic datasets (LS3D-W Balanced and FFHQ) and two real-world datasets (Widerface and WebFace).

**Table 3:** Summary of quantitative results of our proposed approach and other top-performing approaches across the synthetic test datasets (LS3D-W Balanced^36^ and FFHQ^45^).To highlight performance rankings, the best, second-best, and third-best outcomes are indicated using italic, bolditalic, and **bold** text, respectively.The lowest scores for FID, KID, and LPIPS metrics denote superior outcomes while the highest scores for PSNR and SSIM metrics denote superior outcomes.

Method	LS3D-W Balanced^36^					FFHQ^45^				
	FID$\downarrow$	KID$\downarrow$	LPIPS$\downarrow$	PSNR$\uparrow$	SSIM$\uparrow$	FID$\downarrow$	KID$\downarrow$	LPIPS$\downarrow$	PSNR$\uparrow$	SSIM$\uparrow$
Bicuibic Interpolation	154.73	$14.30\pm 0.13$	0.5382	*10.2138*	*0.1153*	139.07	$15.44\pm 0.18$	0.4221	17.2385	0.4938
Hiface-GAN^60^	209.16	$19.18\pm 0.20$	0.5508	9.3872	0.0901	200.11	$20.68\pm 0.30$	0.4544	16.2584	0.4671
Real-ESRGAN^61^	70.04	${\textbf {3.05}}\pm {\textbf {0.08}}$	0.4835	9.4251	0.0980	49.74	$3.08\pm 0.10$	0.2934	17.3180	0.5779
GFPGAN^28^	132.96	$10.99\pm 0.11$	0.5333	***10.1745***	***0.1106***	117.41	$11.95\pm 0.15$	0.3892	**18.5460**	**0.6114**
LRGAN^29^	**58.98**	$3.61\pm 0.11$	**0.4542**	9.6108	**0.0944**	**40.51**	${\textbf {2.86}}\pm {\textbf {0.12}}$	**0.2532**	18.1806	0.5903
PULSE^27^	86.85	$7.56\pm 0.19$	0.4715	**10.0343**	0.0888	80.75	$7.8\pm 0.23$	0.3399	16.4490	0.4479
SCGAN^34^	*34.38*	*1.22*$\pm$*0.06*	***0.4476***	9.3727	0.0889	*19.30*	*0.97*$\pm$*0.05*	***0.1963***	***19.2032***	***0.6847***
Ours	***37.62***	***1.53***$\pm$***0.08***	*0.4443*	9.5325	0.0900	***22.48***	***1.26***$\pm$***0.06***	*0.1896*	*19.4048*	*0.6875*

**Table 4:** Summary of quantitative results of our proposed approach and other top-performing approaches across the real-world test datasets (Widerface^54^ and WebFace^34^).To highlight performance rankings, the best, second-best, and third-best outcomes are indicated using italic, bolditalic, and **bold** text, respectively. The lowest scores for FID, KID, and NIQE metrics denote superior outcomes.

Method	Widerface^54^			WebFace^34^		
	FID$\downarrow$	KID$\downarrow$	NIQE$\downarrow$	FID$\downarrow$	KID$\downarrow$	NIQE$\downarrow$
Bicuibic Interpolation	145.99	$15.40\pm 0.20$	4.9673	166.05	$17.08\pm 0.16$	5.0428
Hiface-GAN^60^	205.32	$20.45\pm 0.27$	2.1923	220.17	$21.01\pm 0.24$	2.3188
Real-ESRGAN^61^	50.93	${\textbf {2.76}}\pm {\textbf {0.09}}$	**2.0464**	67.15	${\textbf {3.54}}\pm {\textbf {0.09}}$	2.0929
GFPGAN^28^	122.61	$11.58\pm 0.17$	4.8298	140.37	$12.98\pm 0.13$	4.9183
LRGAN^29^	**45.45**	$3.09\pm 0.12$	2.2252	**58.60**	$3.64\pm 0.13$	**2.2803**
PULSE^27^	79.97	$7.54\pm 0.20$	3.6331	89.73	$7.79\pm 0.21$	3.5728
SCGAN^34^	*24.85*	*1.08*$\pm$*0.06*	***1.8787***	*35.73*	*1.38*$\pm$*0.053*	***1.8820***
Ours	***26.46***	***1.23***$\pm$***0.07***	*1.6279*	***39.11***	***1.70***$\pm$***0.07***	*1.810*

For the quantitative comparison, we used FID and Kernel Inception Distance KID for both synthetic and real-world datasets. Additionally, we evaluated LPIPS, PSNR and SSIM for synthetic datasets and adopted NIQE for real-world datasets. Table 3 encapsulates the quantitative results across the synthetic test datasets (LS3D-W Balanced and FFHQ), while Table 4 summarizes the quantitative results across the real-world test datasets (Widerface and WebFace). Our method achieves the lowest LPIPS scores on both the FFHQ and LS3D-W Balanced datasets, indicating strong perceptual fidelity and close alignment with human visual perception. While it also attains the highest PSNR and SSIM values on the FFHQ dataset, it does not consistently rank first on LS3D-W Balanced. This result is expected, as PSNR and SSIM tend to favor smooth, pixel-aligned reconstructions and are less suitable for evaluating perceptual quality under significant pose and appearance variations–conditions^78^ that our method is explicitly designed to handle. In terms of distribution-based metrics, our approach achieves the second-best performance on both FID and KID, ranking just behind SCGAN^34^. Furthermore, our framework attains the lowest NIQE score, demonstrating its ability to produce face images that appear more visually realistic and natural compared to other methods. As evidenced by several studies,e.g^65^., FID (and similarly KID) serve as quantitative distributional measures, whereas LPIPS and NIQE, despite yielding numerical scores, are primarily intended to capture perceptual quality and human visual experience,in turn, this enhancement resulted in higher facial verification and detection accuracy, as presented later in Assessment on downstream vision tasks subsection. This is a strong indication that our model excels in constructing high-quality face images without unnatural distortions.

In addition to perceptual metrics, we report the average inference time per image in Table 5. Our method processes a single $64\times 64$ image in just 6.95 ms, achieving the fastest inference among comparable GAN-based face super-resolution approaches while maintaining high perceptual quality.

**Table 5:** Average inference time per image.

Method	Average Inference Time (ms)
HifaceGAN^60^	244.21
Real-ESRGAN^61^	77.72
GFPGAN^28^	305.18
LRGAN^29^	**17.67**
PULSE^27^	21147.53
SCGAN^34^	***14.86***
Ours	*6.95*

**Fig. 4:**
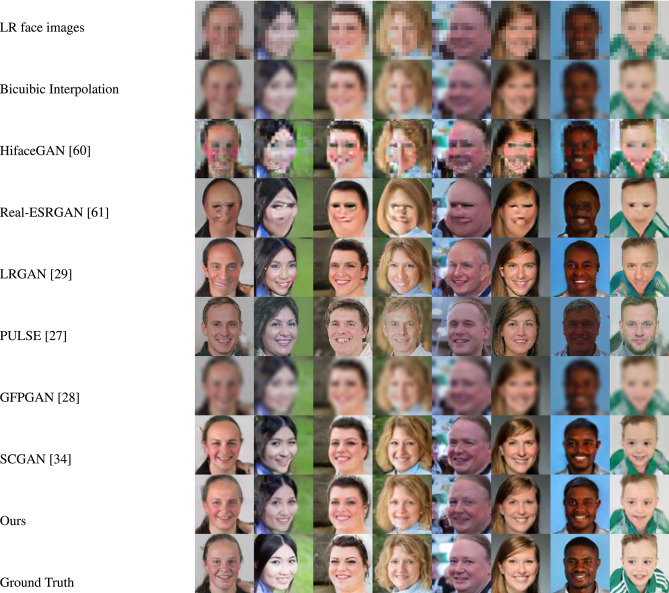
Comparison of visual quality by our approach and other face SR/real-world face SR methods on FFHQ^45^ dataset. For improved clarity, the figure is best examined at higher magnification.

**Fig. 5:**
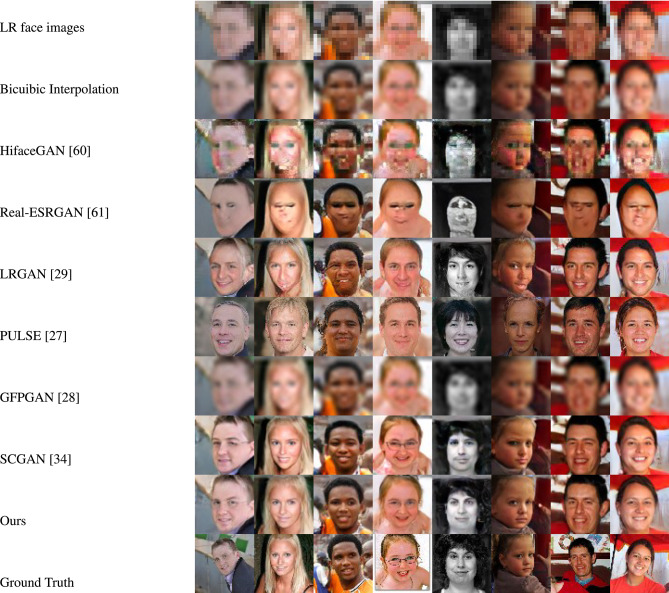
Comparison of visual quality by our approach and other face SR/real-world face SR methods on LS3D-W balanced^36^ dataset. For improved clarity, the figure is best examined at higher magnification.

**Fig. 6:**
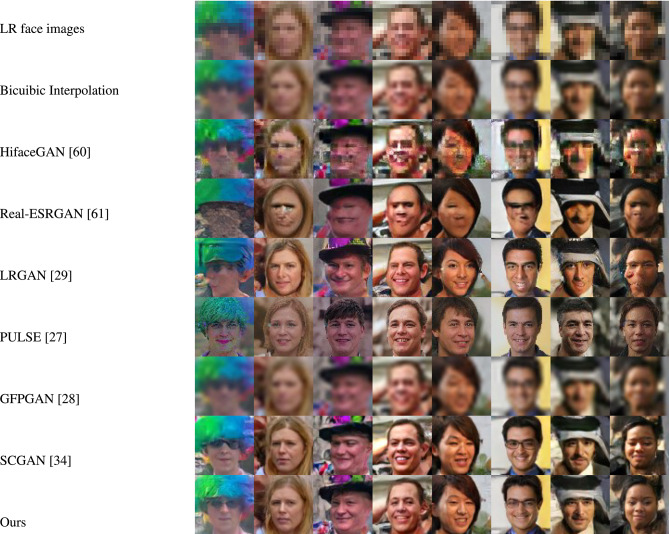
Comparison of visual quality by our approach and other face SR/real-world face SR methods on Widerface^54^ dataset. For improved clarity, the figure is best examined at higher magnification.

**Fig. 7:**
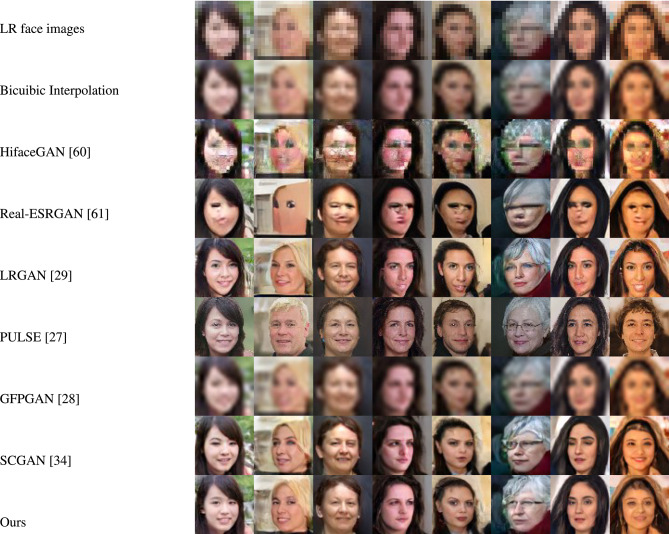
Comparison of visual quality by our approach and other face SR/real-world face SR methods on WebFace^34^ dataset. For improved clarity, the figure is best examined at higher magnification.

Figures 4 and 5 showcase the qualitative evaluation of visual quality across the synthetic test datasets (FFHQ and LS3D-W Balanced), while Figures 6 and 7 are for the real-world test datasets (Widerface and WebFace) respectively. Based on Figs. 4 and 5, it can be concluded inferred that our approach delivers high-fidelity reconstructions, effectively preserving both the structural layout and fine-grained details present in the reference high-resolution facial images. As for the real-world test datasets (Widerface and WebFace) and by examining Figs. 6 and 7, one can deduce that our proposed approach demonstrates a strong capability in recovering facial structure and subtle features.

### Assessment on downstream vision tasks

This section is devoted to assessing the performance of our proposed method alongside other top-performing face super-resolution approaches on various downstream vision tasks, including face detection, and face verification.

#### Face detection

Our approach is evaluated against Bicubic Interpolation, HiFaceGAN^60^, LRGAN^29^, GFPGAN^28^, and SCGAN^34^ using a pre-trained HOG+SVM face detection model^59,62^, and^63^. Face detection accuracy is evaluated across several datasets, as presented in Table 6. Our approach demonstrates superior performance over the competing approaches, as measured by the proportion of images in which faces are correctly detected with bounding boxes, assuming each image contains one face. This superior performance demonstrates the ability of our framework in restoring and preserving facial structures, ensuring that critical facial features are clearly detectable.

**Table 6:** Face detection accuracy on the SR face images results restored by our approach and other methods on the synthetic and real-world test. To highlight performance rankings, the best, second-best, and third-best outcomes are indicated using italic, bolditalic, and **bold** text, respectively.

Method	LS3D-W Balanced^36^	FFHQ^45^	Widerface^54^	WebFace^34^
Bicubic Interpolation	57.60%	48.12%	52.90%	48.05%
HifaceGAN^60^	41.80%	39.08%	40.55%	34.43%
Real-ESRGAN^61^	60.90%	63.48%	57.05%	54.28%
GFPGAN^28^	**93.40%**	90.44%	91.80%	89.59%
LRGAN^29^	92.60%	92.68%	**93.80%**	**92.60%**
PULSE^27^	***96.00%***	**93.24%**	84.55%	76.07%
SCGAN^34^	*97.80%*	*96.84%*	***96.90%***	***96.69%***
Ours	*97.80%*	***95.96%***	*97.40%*	*97.08%*

**Table 7:** Verification Accuracy of FaceNet^64^ on the SR face images in synthetic FFHQ test set^45^ restored by different methods. To highlight performance rankings, the best, second-best, and third-best outcomes are indicated using italic, bolditalic, and **bold** text, respectively.

Method	Accuracy
Bicubic Interpolation	22.92%
HifaceGAN^60^	27.00%
GFPGAN^28^	**74.56%**
LRGAN^29^	56.28%
SCGAN^34^	***85.08%***
Ours	*91.72%*
Ground Truth	97.56%

#### Face verification

Face verification involves determining if two facial images correspond to the same person. As demonstrated in Table 7, our proposed method exhibits strong identity preservation capabilities, particularly when restoring images from the FFHQ test set^45^. Verification experiments using FaceNet^64^ confirm that our proposed method superpasses other methods in maintaining identity-related features, resulting in improved verification accuracy.

## Conclusion

To address the degradation in face recognition performance caused by low-resolution real-world face images, this study strives to advance the quality of such images by integrating facial structural information into a semi-cycle generative adversarial network (SCGAN). The study was built on the idea of combining the GAN’s capability to generate high-quality images that reconcile the domain discrepancy between real-world and synthetic data with the alignment network’s ability to map facial landmarks through heatmap regression and loss optimization. The experimental findings indicate that our proposed method surpasses the forefront methods in terms of perceptual quality and naturalness. This, in turn, contributes to higher-quality face detection and recognition from real-world low-resolution images.

## Data Availability

During our study we used four datasets which are available in the [SCGAN] repository, https://github.com/HaoHou-98/SCGAN. We used four datasets, here are their original sources links: 1. FFHQ dataset is publicly available at: https://github.com/NVlabs/ffhq-dataset. 2. LS3D-W balanced dataset is publicly available at: https://www.adrianbulat.com/face-alignment. 3. WiderFace dataset is publicly available at: http://shuoyang1213.me/WIDERFACE/. 4. WebFace dataset is publicly available by “Semi-cycled generative adversarial networks for real-world face super-resolution” at: https://github.com/HaoHou-98/SCGAN.
